# Mobile Technologies for Supporting Mental Health in Youths: Scoping Review of Effectiveness, Limitations, and Inclusivity

**DOI:** 10.2196/46949

**Published:** 2023-08-23

**Authors:** Shannon Grace Litke, Annie Resnikoff, Ashley Anil, Meredith Montgomery, Rishabh Matta, Jina Huh-Yoo, Brian P Daly

**Affiliations:** 1 Department of Psychological and Brain Sciences Drexel University Philadelphia, PA United States; 2 Department of Information Science Drexel University Philadelphia, PA United States

**Keywords:** mHealth, mobile app, children, adolescents, mental health, effectiveness, efficacy, scoping review, mobile phone

## Abstract

**Background:**

Over the past decade, there has been growing support for the use of mobile health (mHealth) technologies to improve the availability of mental health interventions. While mHealth is a promising tool for improving access to interventions, research on the effectiveness and efficacy of mHealth apps for youths is limited, particularly for underrepresented populations, including youths of color and economically marginalized youths.

**Objective:**

This scoping review study sought to evaluate the following research questions: (1) What is the extent of the current literature on mHealth apps that provide intervention for mental health problems in children and adolescents? (2) What is known from the existing literature about the effectiveness or efficacy of delivering mental health services via mHealth apps? (3) What are the gaps in the knowledge base in the fields of technology and mental health? (4) Do the reviewed mHealth apps address issues of cultural sensitivity or have they been tested with underrepresented groups (ie, youths of color or economically marginalized groups)?

**Methods:**

An electronic database search was conducted using relevant search terms. Seven independent reviewers screened identified studies, including title and abstract review to determine if studies met the following inclusion criteria: (1) targeted samples with mental health symptomology or disorders, (2) studied youth participants aged 6-17 years, and (3) examined the use of a mobile app–based platform for intervention. Relevant studies were subjected to full-text review to extract and chart relevant data based on a priori research questions.

**Results:**

The initial database search yielded 304 papers published from 2010 to 2021. After screening and selection, the final review included 10 papers on the effectiveness and efficacy of mental health intervention apps for youths aged 8 to 17 years. Identified apps targeted a broad range of mental health challenges in youths (ie, depression, self-harm, autism spectrum disorder, anxiety, and obsessive-compulsive disorder). Results identified only a small number of studies suggesting that current effectiveness and efficacy research in this area are limited. While some studies provided general support for the effectiveness of mHealth apps in improving mental health outcomes in youths, several notable limitations were present across the literature, reducing the generalizability of findings. Additionally, considerations around racial, ethnic, and socioeconomic diversity were scarce across studies.

**Conclusions:**

Although some studies cited in this scoping review provide support for the effectiveness and efficacy of mHealth apps targeting mental health concerns in youths, the overall body of literature remains quite limited. Moreover, mHealth apps expressly developed to be culturally responsive are almost nonexistent. Further efforts are needed to recruit youths who are typically underrepresented in research and invite stakeholder participation and collaborative input in the early stages of the mHealth app development process.

## Introduction

Approximately 35% of youths in the United States will have been diagnosed with at least 1 mental health disorder by the time they reach adolescence [1]. When these children do not receive mental health services during the school-age years, the disorders tend to persist [2] and are associated with considerable social, behavioral, and educational and vocational problems and lower quality of life in adulthood [3]. Mobile health (mHealth) is regarded as an important new tool for the assessment and treatment of mental and physical health conditions because these technologies can help reduce logistical and system-level barriers as well as stigma for children and families seeking resources. “mHealth” is a broad umbrella description that refers to various mobile and wireless apps, including SMS text messaging, apps, wearable devices, remote sensing, and the use of social media in the delivery of health-related services [4]. In this paper, the term “mHealth apps” is used to describe mental health mobile technology apps designed specifically for the treatment of mental health symptoms.

Over the previous 2 decades, there has been a sharp increase in the development and use of mHealth apps to improve remote access to, and delivery of, evidence-based care [5] with over 10,000 apps designed specifically for mental or behavioral health available in the marketplace [6,7]. However, few rigorous studies have examined the effectiveness of mHealth apps for youths [8]. In a systematic review of mental health mobile apps for youths [9], authors found insufficient research evidence to support the effectiveness of mHealth apps for youths with mental health problems. Importantly, mHealth’s efficacy in marginalized youth populations has received even less attention in the empirical literature, even though an mHealth model confers unique advantages, including improved access to mental health care, equity of resources, immediate availability, lower cost, and tailored content [9,10]. Given how quickly mobile technologies evolve and are adopted, it is important to constantly examine data from well-designed studies to support practitioners’ understanding around potential benefits and obstacles to the use and effectiveness of mHealth technologies, including with marginalized youth populations.

The familiarity of smartphone apps, ease of use [11], and near ubiquity among youths [12] suggest that mental health treatment providers may benefit from incorporating mHealth technologies and associated features into treatment strategies and plans. The use of mHealth apps can also improve treatment engagement and quality of care by providing more continuous access to self-guided tools [8,13]. For example, mHealth apps can provide easy and timely access to evidence-based strategies that have been demonstrated to reduce mental health symptoms such as journaling, self-monitoring, use of thought records, and relaxation training through increased awareness of the behaviors and practice of these skills [8]. mHealth apps can also promote engagement with these activities through in-app reminders, instructions, and activity templates [14].

While findings from some studies suggest that mHealth apps for mental health are associated with reduced symptoms of depression and anxiety, reduced frequency of self-harm, and increased coping self-efficacy [15], further research on effectiveness is required [9,16]. For example, many reviews cite the inadequacy of previous research in the field, which has ultimately hampered the wider acceptance of mHealth interventions for mental health in youths. These limitations led to recent calls for methodologically robust studies evaluating the safety, efficacy, and effectiveness of these apps [9,17-20]. Furthermore, considering the socioeconomic “digital divide” that may stymie delivery of care to marginalized populations via an mHealth model [21], it is important that research efforts also seek to examine effectiveness within diverse, representative samples of youths.

Underrepresented groups, including youths of color and economically marginalized youths, are at an increased risk for adverse experiences and stressors, compounded by barriers including poor access to care and substandard mental health treatment [22]. Reduced access to care is further compounded by evidence suggesting that the stigma of mental health problems is far greater among youths of color compared to White youths [23,24]. Service pathways and barriers to accessing mental health care are influenced by many individual, interpersonal, and systemic factors [25,26]. Specifically, poor cross-cultural understanding and communication can lead to decreased detection of mental health problems among youths of color [27]. Disparities in connecting with and completing intervention services may remain present even when needs are identified [28]. Adding to this challenge, distrust of mental health professionals, concerns of stigma, or difficulty identifying and accessing quality care, potentially due to lack of resources (eg, shortage of insurance or transportation), may prevent underserved youths from seeking or locating care [29]. A meta-analysis by Hall and colleagues [30] indicated that culturally adapted interventions were associated with high levels of acceptance and satisfaction, in addition to improved outcomes. However, interventions for children that focus on general symptoms of depression, anxiety, and resiliency without inclusion of cultural considerations are more commonplace [31]. Given the widespread use of mobile phones, mHealth has the capacity to provide effective care for vulnerable populations, irrespective of age, socioeconomic status, or geographic area. Health communication technologies can be tailored to fit specific needs in a culturally competent manner to the target population, which is crucial in developing effective interventions.

The objective of this scoping review study was to evaluate the current literature on the effectiveness and efficacy of mHealth apps that target mental health problems in youths. Additionally, this study aimed to identify gaps in the current literature, particularly with regard to including members of underrepresented populations in effectiveness research for mHealth apps designed for mental health treatment in youths.

## Methods

### Study Design

As defined by Arksey and O’Malley [32], a scoping review serves to map the key concepts involved in a particular research area by summarizing research findings and identifying gaps in the evidence base. Compared to a systematic review, which often includes a more narrowly defined research question, a scoping review approach is better suited for characterizing themes from research areas with preliminary evidence or emerging literature, such as the topic of mHealth apps for mental health intervention in youths. To ensure methodological rigor, this review was designed and conducted in accordance with the PRISMA-ScR (Preferred Reporting Items for Systematic Reviews and Meta-Analyses Extension for Scoping Reviews) guidelines [33]. Additionally, the methodological framework set forth by Arksey and O’Malley [32] was used to conceptualize the development of research questions, search and selection procedures, and qualitative analysis as detailed in the following steps.

### Identification of Research Questions

The aim of this scoping review was to identify and synthesize information from the literature according to the following a priori research questions: (1) What is the extent of published evidence on using mHealth apps that provide intervention for mental health problems in children and adolescents? (2) What is known from the existing literature about the effectiveness or efficacy of delivering mental health services via mHealth apps? (3) What are the gaps in the knowledge base in the fields of technology and mental health? (4) Do the reviewed mHealth apps address issues of cultural sensitivity or have they been tested with diverse samples? To explore this final research question in more depth, we examined the following questions: (1) Were there specific considerations in recruitment efforts to include underrepresented groups (ie, youths of color or economically marginalized groups)? (2) For studies that included underrepresented or underserved youths, what did the results suggest were barriers and facilitators to effective use of the mHealth app?

### Identification of Relevant Studies

A search of the APA PsycINFO electronic database was conducted on July 21, 2021, using the following terms: “mental health” OR “counseling” OR “psychotherapy” OR “anxiety” OR “depression” OR “CBT” OR “cognitive behavioral”) AND (“app” OR “app-based” OR “mobile app” OR “smartphone” OR “mobile application”) AND (“children” OR “adolescents” OR “youth” OR “teenagers” OR “high school.” Papers were included in the initial search if they were peer-reviewed, available in the English language, and published between January 2010 and the date of search. The year 2010 was chosen because the literature examining technology-based mental health interventions before this time largely focused on web-based programs that lacked mobile app components [34].

### Study Selection and Screening Procedures

The study selection procedure included two review stages: (1) title and abstract review and (2) full-text review. First, 7 independent reviewers evenly divided the search results to complete a screening of the title and abstract for each paper. Papers were marked as relevant for inclusion in the full-text review stage if they met the following criteria: (1) targeted samples with mental health symptomology or disorders, (2) studied youth participants aged 6-17 years, and (3) examined the use of a mobile app–based platform for intervention. Of note, studies were excluded if they examined nonmobile technologies (eg, a computer-based platform vs a smartphone app). The age range of 6-17 years was selected to best capture the elementary and secondary school-age range of first grade through high school. In addition, the selected age range aligns with inclusion age criteria for the National Survey of Children’s Health study that looked at mental health disorders and disparities of mental health care use in children [35]. Moreover, the age range of 6-17 years is commonly used in cross-cutting symptom measures for youths [36].

To assess the interrater reliability of study selection, a total of 82 papers (20% of total search results) were randomly selected for review by 2 independent reviewers, and the consensus was evaluated by a third reviewer. After full-text review, papers were excluded if they did not directly assess and report outcome data on the effectiveness of the mobile intervention. For example, studies reporting solely on usability, feasibility, or qualitative measures of acceptability were excluded from the final review. Review papers such as meta-analyses, systematic, or scoping reviews were excluded from the final analysis. However, review papers were screened to identify relevant references, which were then entered into the title and abstract review stage for evaluation.

### Charting the Data

After a full-text review, the following data were extracted from papers within the final review pool: sample characteristics (ie, size, age range, clinical presentation, or mental health characteristics), name, purpose, and brief description of the mHealth app, demographic characteristics of the sample, outcome measures, and effectiveness findings.

## Results

### Overview

**Figure 1:**
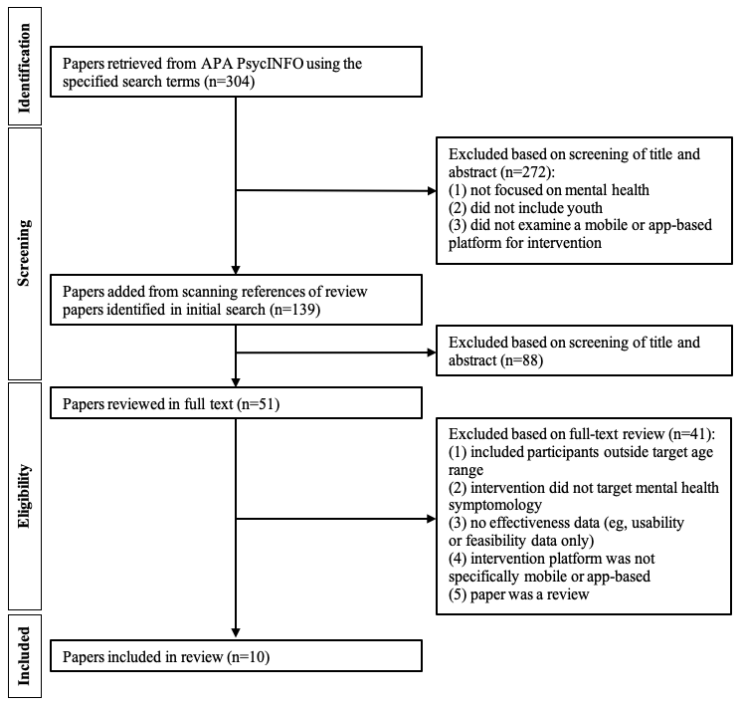
PRISMA-ScR (Preferred Reporting Items for Systematic Reviews and Meta-Analyses Extension for Scoping Reviews) flowchart of study selection.

As displayed in Figure 1, the initial PsycINFO search yielded 304 paper references for further eligibility analysis. Upon title and abstract review, 272 papers were excluded based on the specified criteria. No discrepancies in reviewer’s inclusion or exclusion decisions were noted upon consensus review. Of the papers identified from the initial search that were marked as relevant for full-text analysis (n=32), 13 were review papers (eg, meta-analyses, systematic, or scoping reviews), which were excluded from the final analysis. Screening of the references in these review papers resulted in an additional 107 papers that were examined via title and abstract review, after which 88 were excluded. A total of 51 papers were subject to full-text review, 41 of which were excluded because they included participants outside of the target 6-17 years age range, did not target mental health symptomology, reported no effectiveness data, did not specifically include a mobile- or app-based platform for intervention, or were review papers. The final analysis was conducted on 10 papers. Table 1 presents a summary of the study characteristics.

**Table 1:** Summary of effectiveness studies on mobile interventions for mental health in youths.

App name	Sample	App components	Demographic characteristics	Primary outcome measures	Results
MOSOCO^a^ [37]	N=12; 8-to 11-year-old youths; 25% (n=3) with ASD^b^	Social skills training	No gender, racial or ethnic, or socioeconomic demographic data reported	Quantitative coding of target behaviors from video recordings	76.5% reduction in time engaged in social missteps for students with autism using MOSOCO compared to those not using the app (*P*=.002).
CARE mobile app [38]	N=80; 12- to 15-year-old youths with moderate to high rumination	Mindfulness exercises	53.8% boys, 45% girls, 1.2% chose not to answer; 86.25% White, 2.5% Native American, 1.25% Black, 1.25% multiracial, 8.75% chose not to answer; 93.75% non-Hispanic, 3.75% Hispanic, 2.5% chose not to answer; median parental-reported income range US $100,000-US $125,000; 4% reported recipients of government-assisted food program	MASC,^c^ CRSQ,^d^ CDI,^e^ PSC-I^f^	Significant reduction in rumination (η^2^=0.112), anxiety (η^2^=0.145), and parent-reported internalizing symptoms (η^2^=0.370) at 12-week follow-up.
Open Autism Software suite (apps for tablets)––Drawing, Music, Untangle, Photogoo [39]	N=8; 10- to 14-year-old youths with ASD	Social skills training	62.5% boys, 37.5% girls; no racial or ethnic or socioeconomic demographic data reported	Quantitative coding of target behaviors from video recordings	Significantly more verbal (*P*=.001) and physical (*P*<.05) interactions per minute when using app versus not using app. No difference in number of supportive comments, social missteps, or atypical behaviors.
ICBT BiP OCD^g^ [40]	N=67; 12- to 17-year-old youths with OCD^h^	Web-based CBT,^i^ exposure tasks, ERP^j^ reminders	No gender data reported; 93% born in Sweden, 4% born in other European countries, 3% Asian; no socioeconomic demographic data reported	CY-BOCS^k^	Significantly greater reduction in CY-BOCS scores over time for youths using BiP OCD^l^ intervention compared to waitlist control at posttreatment (*d*=0.69) and 3-month follow-up (*d*=1.68).
BYOTS^m^ [41]	N=72; 12- to 15-year-old youths	Cognitive restructuring	100% female; 100% Black or biracial; 100% enrolled in the federal free breakfast and lunch program	MASC	Significant reduction in MASC total anxiety scores from pre- to postintervention (*d*=0.52).
WeClick [42]	N=193; 12- to 16-year-old youths	Cognitive restructuring, social learning, problem-solving, and conflict resolution skill development	86.53% female; 3.6% Aboriginal or Torres Strait Islander; no socioeconomic demographic data reported	PHQ-A,^n^ WEMWS,^o^ GHSQ^p^	Decrease in PHQ-A scores from baseline to posttest was not significant (*P*=.138). Significantly greater increase in select secondary outcomes for app users versus control, including well-being (WEMWS; *d*=0.37), help-seeking intention (GHSQ; *d*=0.36), and professional help-seeking intention (GHSQ-P^q^; *d*=0.36).
SmartCAT (version 2.0) [43]	N=34; 9- to 14-year-old youths with anxiety (GAD,^r^ SAD,^s^ or SocAD^t^)	CBT, skill development, and exposure tasks	50% female; 85.3% White, 2.9% Hispanic, 14.7% biracial; total family income mean US $70,001-US $90,000	PARS^u^	Significant reduction in PARS severity scores from pre- to posttreatment (*d*=1.05) and posttreatment to 2-month follow-up (*d*=0.55).
BlueIce [20]	N=44; 12- to 17-year-old youths with current or past self-harm	CBT and DBT^v^ harm-reduction strategies, safety checks, and rerouting to emergency services when necessary	91% girls; no racial or socioeconomic demographic data reported	SDQ,^w^ MFQ,^x^ RCADS^y^	Reduction in self-harm in 73% of participants as well as a significant reduction in depression (MFQ; *P*=.04) and anxiety (RCADS; *P*=.001) symptoms postintervention.
Mayo Clinic Anxiety Coach [44]	N=2; 10- and 16-year-old youths with OCD (case study)	Exposure tasks, ERP	10-year-old White female, 16-year-old White male; no socioeconomic demographic data reported	CY-BOCS	Decrease in OCD severity ratings from pre- to posttreatment was observed for both participants in this case study.
Mayo Clinic Anxiety Coach [45]	N=8; 8- to 17-year-old youths with a primary diagnosis of an anxiety disorder or OCD	Exposure tasks, ERP	75% girls; 100% White; no socioeconomic demographic data reported	PARS, SCAS,^z^ CRS,^aa^ CGI-S/I^ab^	Decrease in CGI-S/I, CRS, SCAS, and PARS scores from pre- to posttreatment. Reported large effect sizes.

^a^MOSOCO: Mobile Social Compass.

^b^ASD: autism spectrum disorder.

^c^MASC: Multidimensional Anxiety Scale for Children.

^d^CRSQ: Children's Response Styles Questionnaire.

^e^CDI: Children's Depression Inventory.

^f^PSC-I: Pediatric Symptom Checklist-Internalizing Subscale.

^g^ICBT BiP OCD: Internet-delivered CBT BarnInternetProjektet for obsessive-compulsive disorder.

^h^OCD: obsessive-compulsive disorder.

^i^CBT: cognitive behavioral therapy.

^j^ERP: exposure and response prevention.

^k^CY-BOCS: Children’s Yale-Brown Obsessive-Compulsive Scale.

^l^BiP OCD: BarnInternetProjektet for obsessive-compulsive disorder.

^m^BYOTS: Build Your Own Theme Song.

^n^PHQ-A: Patient Health Questionnaire-Adolescents.

^o^WEMWS: Warwick Edinburg Mental Wellbeing Scale.

^p^GHSQ: General Help-Seeking Questionnaire.

^q^GHSQ-P: General Help-Seeking Questionnaire—Professional Help-Seeking Intentions Score.

^r^GAD: generalized anxiety disorder.

^s^SAD: separation anxiety disorder.

^t^SocAD: social anxiety disorder.

^u^PARS: Pediatric Anxiety Rating Scale.

^v^DBT: dialectical behavioral therapy.

^w^SDQ: Strength and Difficulties Questionnaire.

^x^MFQ: Mood and Feelings Questionnaire.

^y^RCADS: Revised Child Anxiety and Depression Scale.

^z^SCAS: Spence Children’s Anxiety Scale.

^aa^CRS: clinician’s severity rating.

^ab^CGI-S/I: Clinical Global Impression Scale—Severity/Improvement.

### Exploring Research Aim 1: Extent of the Literature

#### Overview

Overall, results of this scoping review reveal that efficacy and effectiveness research on mobile platforms targeting mental health concerns in youths are limited with only a small number of studies examining intervention apps (N=10). It is important to highlight that the paucity of studies in this area renders it inappropriate to draw overarching conclusions about the efficacy or effectiveness of app-based interventions for mental health problems in youths. Although limited in number, the mHealth apps identified across these studies use a variety of innovative strategies for managing mental health concerns that are worthy of description. Participants in these studies ranged from age 8 to 17 years and presented with a variety of clinical symptomatology and disorders, including depression and self-injurious behavior [20], autism spectrum disorder (ASD) [37,39], anxiety [43], and obsessive-compulsive disorder (OCD) [40,44,45].

Additionally, 2 studies [38,41] examined mobile apps targeting symptoms of anxiety, although they included school-based or community (rather than clinical) samples of youths. Furthermore, one study reported on a mobile app, “WeClick,” (Black Dog Institute) designed to improve overall well-being for youths with a broad range of mental health problems [42] but with a specific focus on the primary outcome of depressive symptoms. The next section describes the apps included in the scoping review.

#### Depression and Self-Harm

This review identified one effectiveness study of an app specifically targeting depression and self-harmful behavior in adolescents. BlueIce (Oxford Health NHS Foundation Trust and University of Bath) is a smartphone app designed to provide youths who have a history of self-harm with 24/7 access to a variety of cognitive behavioral therapy (CBT) and dialectical behavioral therapy intervention tools [20]. Strategies included identifying and challenging negative cognitions, safety planning and routing to appropriate emergency contacts or services, distress tolerance tools, and a menu of personalized activities designed to improve mood [20].

Additionally, the WeClick app [42] was designed as a single-session intervention based on principles of CBT and social learning theory. Specifically, this mobile intervention focuses on relationship-specific issues (eg, family and peer conflict, intimate relationships, and substance use) and promotes cognitive restructuring to help youths overcome these relationship challenges.

#### Autism Spectrum Disorder

A study by Hourcade and colleagues [39] describes a package of tablet apps available through the Open Autism Software suite (University of Iowa) designed as a mobile intervention to promote social interaction in youths with ASD. Specifically, the apps include activities to encourage face-to-face social interaction and enhance creativity, sharing, turn-taking, and emotion modeling [39]. Similarly, the Mobile Social Compass app (Social & Technological Action Research Group at the University of California, Irvine) was designed to support social skills practice in real-life situations for youths with ASD [37]. This mobile-assistive technology uses the smartphone camera to augment real-life social situations (eg, interaction on the playground at recess) with on-screen visual supports and reminders (eg, cues to make eye contact with the conversation partner). As both platforms highlight, the functionality allowed by mobile intervention modalities (vs table-top or computer-based) offers a unique benefit to youths with ASD, allowing them to access social-skills coaching in real time throughout the day [37,39].

#### Anxiety

Several app-based interventions targeting anxiety symptoms in youths have been developed and examined for efficacy within the past decade. Silk and colleagues [43] conducted a study examining the SmartCAT (version 2.0; University of Pittsburgh) app, a mobile platform designed as an adjunctive resource to support the practice of CBT skills outside of treatment sessions for youths with anxiety disorders. SmartCAT (version 2.0) uses a web-based interface to engage youths, provide cues and digital rewards for practicing skills at home, and prompt youths to engage in personalized exposures outside of session [43]. A mindfulness mobile app, as described in a recent study by Hilt and Swords [38], specifically targets adolescents’ rumination and worry through a variety of mindfulness techniques, including breathing exercises and body scan activities. Further, Neal-Barnett and colleagues [41] developed and evaluated the Build Your Own Theme Song (BYOTS) app (Kent State University), a mobile app designed to deliver a cognitive restructuring intervention to Black middle-school girls. The BYOTS app incorporates a culturally informed approach involving a musical intervention to help users identify and disrupt negative thought cycles by creating their own personal, affirming theme songs [41].

#### Obsessive-Compulsive Disorder

Lenhard and colleagues [40] evaluated the effectiveness of a clinician- and parent-supported web-based CBT program for OCD, BiP OCD (BarnInternetProjektet for obsessive-compulsive disorder; Barn-och ungdomspsykiatri Region Stockholm), which includes a smartphone app to facilitate the child’s completion of exposure tasks, manage progress, and engage parents in psychoeducation on parent-specific topics (eg, family accommodation). Similarly, the Mayo Clinic Anxiety Coach app (Mayo Clinic) [44,45] facilitates parent coaching of exposure therapy for youths with OCD and other anxiety disorders outside of therapy sessions. Additionally, this app includes a variety of clinical content, including tabs with graphics and information to support psychoeducation on different topics, direct access to a tool for youths to build and edit their own fear hierarchies, and built-in forms to support the monitoring of anxiety symptoms. Overall, these apps are designed to support youths in applying the skills learned in treatment for OCD by engaging in out-of-session exposure and response prevention, which is empirically supported as the gold-standard behavioral treatment for youths with OCD [46].

### Exploring Research Aim 2: Evidence of Effectiveness and Efficacy

#### Overview

Although few in number, the intervention apps identified in this review were generally found to be associated with symptom reduction and clinical improvement. Interestingly, the handful of mobile interventions targeting anxiety and OCD consistently demonstrated positive effects, while apps targeting other domains of clinical symptomatology (ie, depressive symptoms, self-harm, and ASD) showed more variability in results. Of note, due to the small number of effectiveness and efficacy studies identified in this scoping review, results have limited generalizability and should be interpreted with caution.

#### Anxiety and OCD

A study by Hilt and Swords [38] found that use of the mindfulness app intervention was associated with significant reductions in rumination, as well as other anxiety and internalizing symptoms from pre- to postintervention, with a large effect persisting throughout a 12-week follow-up period. Similarly, Silk and colleagues [43] reported that the SmartCAT (version 2.0) app demonstrated a large effect on reducing anxiety symptom severity from pre- to posttreatment, with a moderate effect persisting at a 2-month follow-up. This change was found to be statistically significant, and clinically meaningful, with 20 out of 30 (66.67%) participants no longer meeting diagnostic criteria for an anxiety disorder at posttreatment [43]. Further, a study by Lenhard and colleagues [40] marks an important contribution to the literature as one of the 2 randomized controlled designs identified in this overall review. Findings revealed that use of the BiP OCD app had a large effect on significantly reducing OCD symptom severity from pre- to posttreatment and at 3-month follow-up [40]. The use of another app targeting OCD and anxiety symptoms, Mayo Clinic Anxiety Coach, was found to be associated with a significant reduction in symptom severity with authors reporting a large effect size; however, conclusions are limited by the small sample sizes in both studies examining this app [44,45]. Additionally, Neal-Barnett and colleagues [41] reported a medium effect size for BYOTS app use, which was associated with significant reductions in total anxiety scores on the Multidimensional Anxiety Scale for Children (MASC) from pre- to postintervention.

#### Depression and Self-Harm

Examination of the 2 identified studies of intervention apps targeting depressive symptoms and self-harm reveals mixed results. Use of BlueIce was associated with significant reductions in depression and anxiety symptoms as well as reduced frequency of self-injurious behavior in youths presenting with current or past self-harm [20]. In contrast, a randomized controlled trial of the WeClick intervention indicated that the use of this platform was not associated with a significant reduction in depressive symptoms, although some secondary outcomes such as mental health well-being and help-seeking intentions showed significant improvement over time [42].

#### Autism Spectrum Disorder

For the 2 identified studies of apps targeting the development of social skills in youths with ASD, findings generally support the use of the app in facilitating positive outcomes; however, there were some differences noted between the apps in terms of the specific outcomes. For instance, use of the Mobile Social Compass intervention was associated with a significant reduction in frequency of social missteps for youths with autism [37]. In contrast, the Open Autism Software suite did not seem to impact social missteps, atypical behaviors, or frequency of supportive comments for youths with autism. However, the authors noted a significant increase in number of verbal and physical interactions per minute while using the app [39]. Of note, data in these studies were limited, consisting of quantitative coding of target behaviors in video recordings. As such, without multimodal data from other empirically validated measures or psychometric standards, results should be interpreted with caution in terms of the effectiveness of these apps.

### Exploring Research Aim 3: Limitations in the Literature

The mobile intervention apps identified in this review were generally found to have positive effects on mental health outcomes in youths; however, there were several notable limitations among these studies that reduce the generalizability of findings. First, many of the studies included in this review were limited by small sample sizes [37,39,44,45]. Of the larger studies, only 2 were randomized controlled trials [40,42], and the other studies used within-subjects designs to assess intervention effects [20,38,41,43]. As acknowledged by many of these authors, randomized controlled trials mark an important next step in replicating findings from these pilot studies and assessing the effectiveness of these apps while controlling for within-subjects factors.

Most studies in this review involved samples with a limited range of baseline symptom severity, posing an additional threat to generalizability and highlighting an important gap to bridge in future effectiveness studies. For example, results from a study by Hourcade and colleagues [39] suggest that the Open Autism Software suite is a promising skill-development tool for youths with ASD, but the sample was limited to individuals on the high-functioning end of the spectrum. Additionally, Neal-Barnett and colleagues [41] reported that use of the BYOTS app was associated with a statistically significant reduction in the MASC from preintervention to postintervention. While this reduction does mark a shift from “high average” to “average” symptom severity according to cutoff ranges on the MASC [47], it is important to note that participants’ average anxiety severity scores were subclinical at both time points. Therefore, further research is needed in order to determine the effectiveness of this app for youths displaying clinically significant anxiety severity. Similarly, work by Lenhard and colleagues [40] highlights that further investigation of the BiP OCD intervention is needed to identify the clinical appropriateness of this app for youths presenting with greater severity of OCD symptoms. Specifically, the field would benefit from research aimed at pinpointing the symptom profiles of youths that suggest the need for in-person clinical care and to identify whether there is a use case for a stand-alone mobile intervention for a subset of the clinical population whose symptoms are less impairing.

Several authors commented on implications and directions for further research based on gaps in the literature that were left unaddressed in their studies. For instance, Hilt and Swords [38] emphasize the need for further investigation of dosage to understand the minimum amount of user interaction required to achieve meaningful outcomes. Similarly, certain apps were not tested as stand-alone apps but rather in tandem with larger intervention programs [40,41,43-45].

Further dismantling design analyses are required to determine the specific effectiveness or efficacy of these app-based intervention components when used as stand-alone options. Additionally, certain authors [41,43] emphasized the need for continued research on the specific mechanisms of change underlying response to these mHealth interventions as well as investigation of their effectiveness or efficacy across diverse clinical and demographic profiles.

### Exploring Research Aim 4: Inclusion of Underrepresented Groups

Considerations around cultural, ethnic, and socioeconomic diversity were alarmingly scarce across studies included in this scoping review. In addition, the level of detail regarding reported sample demographic information was inconsistent across studies. For example, a handful of studies did not report any demographic data on participants’ race, ethnicity, or socioeconomic status [20,37,39], while other studies reported limited information [40,42]. In the studies that did include details of the racial, ethnic, and socioeconomic characteristics of youth participants, their samples lacked diversity and the authors did not note procedures to recruit members of historically marginalized populations or those underrepresented in research [38,43-45]. Some authors explicitly mentioned that the generalizability of results was limited by a lack of diverse sampling [38], while others did not acknowledge this limitation. Of the 10 studies included in this review, only 1 study included an explicit focus on testing the app in a sample of underserved, underrepresented youths, who were Black adolescent females from economically marginalized backgrounds [41].

Neal-Barnett and colleagues [41] cite a plethora of evidence to support their emphasis on the use of music as a powerful, culturally significant tool for Black youths. For example, these authors discussed the historical importance of gospel and spiritual music for Black adolescents and young adults during the civil rights era as well as the emergence of hip-hop as a mode of expression for Black youths facing marginalization in underserved, urban areas. Drawing on research that shows Black female youths are (1) disproportionally affected by anxiety disorders, (2) clinically underserved and underrepresented in research, and (3) more likely to turn to music to express their emotions than their male counterparts, the authors exclusively recruited a sample of Black or biracial female adolescents from seventh- and eighth-grade classrooms across a low-income, urban school district where 100% of students were economically underserved.

To examine barriers and facilitators relevant to use of the app, qualitative interviews were conducted. Results revealed that girls in the sample were initially skeptical over whether the app would work for them. Their doubts reportedly stemmed from their prior experiences as participants in other research initiatives, wherein the outcomes did not benefit them as they had expected. By the end of the BYOTS study, however, participants reported being pleased that their expectations of the app matched their actual experience, and “it really worked!” [41]. This qualitative finding holds important implications in terms of researchers’ responsibility to build trust and rapport with participants by continuously soliciting participant feedback to collaboratively iterate and improve the user experience. Importantly, these authors acknowledged limitations to generalizability within their own study, namely that Black girls represent a heterogenous population. Further research on the effectiveness of the BYOTS app is needed, including Black females residing in different settings (eg, rural and suburban) and from a variety of socioeconomic status levels [41].

## Discussion

### Principal Findings

While there are a considerable number of effectiveness and efficacy studies of mobile mental health technologies in adult populations [48], findings from this scoping review highlight the paucity of these types of studies relevant to mHealth apps for youths. Instead, much of the current literature is focused on preliminary studies of feasibility or acceptability. For example, a recent study by Punukollu and colleagues [49] examined student and teacher buy-in to the Safespot app (SafeSpot Limited), a mental health support app designed for use in school settings. Qualitative results of this study demonstrated that this app was regarded as acceptable and likable by stakeholders [49]. Although this study was excluded from this review due to the lack of outcome data on the app’s effectiveness, it is important to note that these types of pilot feasibility and acceptability studies often precede larger-scale efficacy and effectiveness trials. As such, the fact that numerous smaller-scale, qualitative studies of mHealth apps are currently underway may be considered a promising marker for future effectiveness trials. However, it is also important to consider that many pilot apps do not reach the efficacy or effectiveness stages of research often due to technological advances outpacing the slower and more deliberate process of psychological research.

From the 10 papers included in this review, several overarching themes emerged, including implications of the narrow range of mental health disorders in the effectiveness studies, considerations surrounding regulation and safety of mHealth technologies, and the limited number of studies that included diverse cultural, ethnic, and socioeconomic samples.

### Narrow Clinical Targets

Most studies with larger-scale effectiveness data targeted anxiety-related disorders. This is perhaps not surprising given that anxiety is one of the 3 most diagnosed mental health disorders in children [50] and is the most diagnosed disorder among 12- to 17-year-olds, an age range more likely to own a smartphone or tablet device as compared to younger children [51]. Even in the adult literature, most of the large-scale effectiveness studies of mHealth apps focus on anxiety and depression [52]. Thus, there is a gap in high-quality effectiveness studies of mHealth apps for other mental health disorders frequently seen in youths including eating disorders, disruptive behavior disorders, and OCD. In the absence of reliable outcome data on the use of mHealth apps targeted at specific disorders, clinicians may rightfully feel cautious in recommending mHealth apps to their clients. Similarly, consumers may struggle to discern which mHealth apps provide benefits relative to their specific mental health challenges.

### Safety Regulations

In recent years, there have been increased regulatory efforts for mobile medical apps that focus on safety and effectiveness; however, groups such as the Food and Drug Administration [53] do not regulate health and wellness apps that are not intended for medical use. Instead, clinicians and consumers can access websites such as PsyberGuide [54] that provide ratings and reviews by mental health experts that include any information on published evidence. Although these types of websites provide rating criteria developed by mental health experts, the proliferation of mHealth apps into the marketplace is so rapid that these websites frequently struggle to keep pace with timely reviews. As an alternative, some organizations have published frameworks that clinicians can use to evaluate mental health apps [55]. Even with these frameworks, safety regulations and guidelines for contraindications of mHealth app use are important targets of future research.

Investigations are also needed that examine individual characteristics of populations for whom mHealth apps prove effective. For example, research suggests that mHealth apps have the potential to act as a preventative tool for individuals with subclinical symptoms or as a maintenance tool for individuals who are no longer in need of traditional professional help but are compelled to continue treatment in a more cost-effective manner [56].

### Recommendations for Addressing Inclusivity

The surprising lack of studies on the use of mHealth apps in underrepresented populations (namely, youths of color and economically marginalized youths) has been observed in many types of digital health apps across all age groups [57-59]. This failure to include diverse samples must be addressed in the development and evaluation of digital health tools, given the systemic inequities facing youths of color with regard to access and bias in receiving quality health care. Review and perspective papers suggest multiple approaches for addressing the gap [57-62], but we focus on three themes that apply to increasing evidence on mental health apps for underrepresented youths: (1) tailorability, (2) human-centered design, and (3) trust-building.

The increased adaptability of digital approaches can enable tailored content and adjust the type or dosage of the intervention based on individual characteristics (eg, multiphase optimization strategy [63] and sequential, multiple assignment, randomized trials) [64]. For youth populations, the solution should be tailored to factors such as the individual’s racial, economic, and educational background, sex, family structure (ie, parenting and guardian contexts), personal hobbies and interests, and types and levels of mentorship available. In other words, digital interventions should be tailored beyond group characteristics (eg, race) to address individual differences.

As more digital mental health apps are developed, incorporating human-centered design and community-centered approaches will help identify the barriers and develop strategies to mitigate those barriers [57,61,65-67]. Recruitment strategies should be culturally sensitive and address community mistrust, participant resource constraints, and potential risks such as community stigma [68]. Participatory design has a long history of effectiveness in addressing community needs [69-72] and is a widely used method in human-computer design for youth populations, given its ability to increase interest and engagement in youths. For example, the BlueIce app [20], one of the mHealth apps included in this review, used a co-design model in the development phase, including a team of youth participants who collaboratively advised on aspects of the app content, design, and layout. Stallard and colleagues [20] found that not only was use of the BlueIce app associated with significant reductions in anxiety, depression, and self-harm but also that this app ranked highly on measures of acceptability with 29 out of 33 (88%) participants indicating that they would like to keep using the app after the study. This type of participatory approach should be adopted as a standard procedure across the development and evaluation of mHealth apps with particular efforts to include underrepresented voices in this process to inform culturally salient adaptations and maximize benefits to recipients of the mHealth intervention.

Even when mental health interventions are available and accessible, trust is still one of the critical barriers to the adoption of interventions by underrepresented populations [68,73]. The process of including end users from the beginning of the design phase, such as participatory design, can increase trust, engagement, and adoption [63,74]. The solution's clinical testing results also should be explicitly available [75] and understandable by the end-user population. Moreover, the solution should have the ability to integrate with existing clinical, community, and school environments to enable broader dissemination and participation.

### Limitations

Many efforts were taken to maximize methodological rigor while conducting this review, but there are several important limitations. First, this review used only 1 database (ie, APA PsycINFO) for the initial search and identification of relevant studies rather than replicating the search across multiple databases. Although APA PsycINFO is widely regarded as a reliable and comprehensive index of records spanning the field of psychological science, it is possible that the initial search conducted for this review did not capture all relevant mHealth studies. Additionally, while reasons for exclusion were documented and categorized as part of the process of the full-text review process for determining eligibility, some studies met several exclusion criteria (eg, participants outside of the specified age range as well as lack of effectiveness data). Due to these overlapping exclusion categories, this analysis lacks a precise, quantitative measure of the number of apps excluded for each specific reason. These data may have offered a more precise understanding of the current volume of feasibility or acceptability studies of mHealth apps for youths. Further, it is important to acknowledge that changes in the technological landscape often occur more rapidly than the pace at which research is conducted, reviewed, and published. It is likely that even the more recent apps highlighted in this review will have markedly evolved or perhaps been discontinued. While it is important to take these limitations into account when interpreting the results and implications of this study, the overall integrity and relevance of this review remain intact. Namely, findings serve to clarify the state of the current literature and prompt critical consideration of future directions for research on mHealth apps for youths with mental health concerns.

### Conclusions

There is a pressing need for evidence-supported adjunct options to mental health therapy services for youths given the clear gap between prevalence and treatment rates. For example, only about one-third of children and adolescents with mental health problems access treatment [76,77], and many encounter pervasive barriers such as lengthy waitlists to see mental health providers, and few providers accept insurance [78]. When considering the ubiquity of technology and the comfort with which youths interface with mobile devices, the promise of mHealth apps has always held the potential to be a supplemental option for mental health support, assessment, and intervention for children and adolescents with mental health concerns. mHealth apps provide easy and real-time accessibility for youths to evidence-based tools and can provide some level of support when stigma or treatment barriers impede access to mental health providers. Although some studies cited in this scoping review provide support for the effectiveness and efficacy of mHealth apps targeting mental health concerns in youths, the overall body of literature remains quite limited. Moreover, mHealth apps expressly developed to be culturally responsive are almost nonexistent. These findings suggest the promise of mHealth apps for mental health in youths remains unfulfilled but is conversely not without optimism.

Increased advocacy and engagement by mental health providers in the development, implementation, and evaluation of mHealth apps would go a long way to inspiring more confidence about effectiveness for end users as well as for treatment providers who are interested in recommending apps as supplemental support options. Further efforts are also needed that focus on recruiting diverse samples, including samples of youths who are typically underrepresented in research, and on being inclusive by inviting participation and collaborative input in the early stages of the mHealth app development process. It takes a village to support mental health in youths, and mHealth can play an important role in the overall support system.

## Abbreviations

ASD: autism spectrum disorder
BiP OCD: BarnInternetProjektet for obsessive-compulsive disorder
BYOTS: Build Your Own Theme Song
CBT: cognitive behavioral therapy
MASC: Multidimensional Anxiety Scale for Children
mHealth: mobile health
OCD: obsessive-compulsive disorder
PRISMA-ScR: Preferred Reporting Items for Systematic Reviews and Meta-Analyses Extension for Scoping Reviews
